# Repetitive Transcranial Magnetic Stimulation Promotes Neural Stem Cell Proliferation via the Regulation of MiR-25 in a Rat Model of Focal Cerebral Ischemia

**DOI:** 10.1371/journal.pone.0109267

**Published:** 2014-10-10

**Authors:** Feng Guo, Xiaohua Han, Jinghui Zhang, Xiuxiu Zhao, Jicheng Lou, Hong Chen, Xiaolin Huang

**Affiliations:** 1 Department of Rehabilitation Medicine, Tongji Hospital, Tongji Medical College, Huazhong University of Science and Technology, Wuhan, China; 2 Department of Rehabilitation Medicine, The Third Affiliated Hospital, Sun Yat-sen University, Guangzhou, China; 3 Department of Obstetrics and Gynecology, Tongji Hospital, Tongji Medical College, Huazhong University of Science and Technology, Wuhan, China; University of Oxford, United Kingdom

## Abstract

Repetitive transcranial magnetic stimulation (rTMS) has increasingly been studied over the past decade to determine whether it has a therapeutic benefit on focal cerebral ischemia. However, the underlying mechanism of rTMS in this process remains unclear. In the current study, we investigated the effects of rTMS on the proliferation of adult neural stem cells (NSCs) and explored microRNAs (miRNAs) that were affected by rTMS. Our data showed that 10 Hz rTMS significantly increased the proliferation of adult NSCs after focal cerebral ischemia in the subventricular zone (SVZ), and the expression of miR-25 was obviously up-regulated in the ischemic cortex after rTMS. p57, an identified miR-25 target gene that regulates factors linked to NSC proliferation, was also evaluated, and it exhibited down-regulation. To further verify the role of miR-25, rats were injected with a single dose of antagomir-25 and were subjected to focal cerebral ischemia followed by rTMS treatment. The results confirmed that miR-25 could be repressed specifically and could drive the up-regulation of its target gene (p57), which resulted in the inhibition of adult NSC proliferation in the SVZ after rTMS. Thus, our studies strongly indicated that 10 Hz rTMS can promote the proliferation of adult NSCs in the SVZ after focal cerebral ischemia by regulating the miR-25/p57 pathway.

## Introduction

The risk of cerebrovascular disease increases substantially with age, making it the leading cause of death and severe long-term disability in humans [1]. The discovery of adult neural stem cells (NSCs) in 1992 brought new life to the treatment of cerebral vascular accidents [2]. It has been found that cerebral ischemia can activate the proliferation of dormant NSCs, accompanied by limited improvement to damaged neurological functions [3], [4]. Thus, exploring methods to promote the proliferation of dormant NSCs after cerebral ischemia may be a promising strategy for ischemic stroke.

Repetitive transcranial magnetic stimulation (rTMS), a technique used to repeatedly, non-invasively induce electric currents in a small area of the brain, has been widely applied in cerebral ischemia therapy recently. The latest research has reported that rTMS promotes the proliferation of adult NSCs in healthy rats; however, knowledge of the underlying mechanism has not yet been defined [5]. Additionally, little work has focused on the effects of rTMS on adult NSC proliferation in cerebral ischemia models, although the mechanism of rTMS in neurogenesis is critical for developing better therapies for cerebral ischemia patients.

MicroRNAs (MiRNAs) are endogenous, short RNA sequences that have long been posited to be involved in neurogenesis and are thought to regulate the expression of target genes by base-pairing with specific binding sites located in the 3′-untranslated region of the target mRNAs [6]. Because of the profound effects that rTMS has on gene expression, it is theoretically possible that they have the potential to regulate miRNA levels. Nevertheless, few reports have attempted to explain how rTMS regulates the proliferation of NSCs via miRNA. Recently, our group found that miR-25 increased significantly after 10 Hz rTMS treatment of cerebral ischemia in rats. This result suggested that 10 Hz rTMS played a regulatory role in the expression of miR-25.

MiR-25 belongs to the miR-106b∼25 cluster. This cluster is located within an intronic region of the Mcm7 gene and codes for three different mature miRNA species: miR-106b, miR-93 and miR-25. Emerging data have indicated that the miR-106b∼25 cluster plays a critical role in adult NSC proliferation [7], [8]. Additionally, Brett et al.'s group reported that the miR-106b∼25 cluster could promote the proliferation of adult NSCs mainly due to miR-25 [9], [10], which also appeared to be one of the strongly expressed miRNAs in the post-ischemic brain [11]. In fact, miR-106b and miR-93 have the same seed sequence and similar 3′-halves, whereas miR-25-lacking the same sequence-is expected to have a separate function [12].

p57 and PTEN were identified as miR-25 target genes [13], [14]. As is known, p57 is a Cip/Kip family member of cyclin-dependent kinase (Cdk) inhibitors that blocks the cycle progression through all stages of G1/S, whereas PTEN is the first phosphatase to regulate G0/G1 cell cycle [15]. These factors suggest that miR-25 may be an important agent in rTMS for promoting adult NSC proliferation after focal cerebral ischemia. Therefore, we hypothesized that rTMS would increase the expression of miR-25 and repress the expression of its targets, thereby promoting adult NSC proliferation and inhibiting cell-cycle arrest after focal cerebral ischemia. To further demonstrate this hypothesis, we preliminarily tested the impact of miR-25 inhibition on its target proteins, investigated the effect of its deletion on the proliferation of adult NSCs with rTMS treatment after focal cerebral ischemia, and sequentially explored associated functions of miR-25 in vivo.

In this study, we found that 10 Hz rTMS could promote the proliferation of NSCs in the SVZ after focal cerebral ischemia, and miR-25 was significantly up-regulated after rTMS treatment, while the proliferation of NSCs in SVZ was blocked by the inhibition of miR-25. All data suggest that miR-25 plays an important role in the therapeutic effects of rTMS on NSC proliferation in the SVZ after focal cerebral ischemia and that rTMS has potential in the rehabilitation of neural function after focal cerebral ischemia.

## Materials and Methods

### Ethics Statement

The experimental designs and all procedures were in accordance with the National Institutes of Health Guide for the Care and Use of Laboratory Animals. All animal experiments were approved by the ethics committee of the Tongji Medical College (Permit Number: 298). The utmost efforts were made to minimize the number of animals used and their sufferings.

### Animals and Experimental Groups

**Figure 1:**
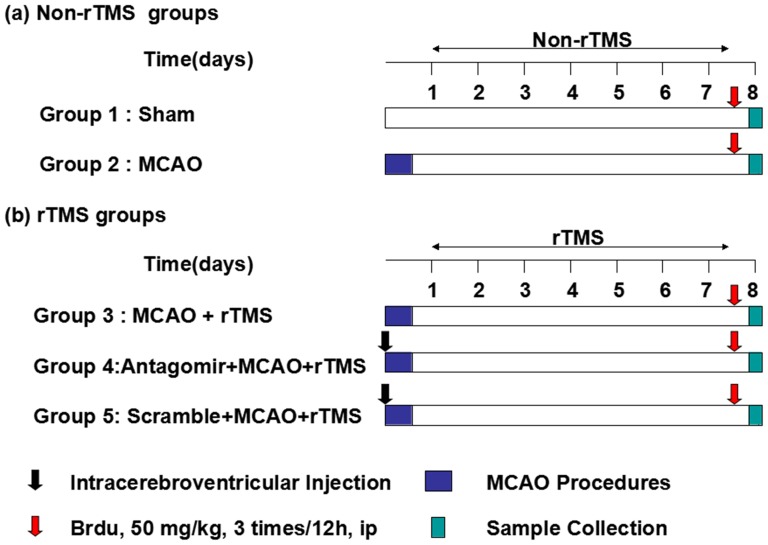
Experimental protocol.

Seven-week-old male Sprague-Dawley rats (weight, 230 to 270 g; Huazhong University of Science and Technology Experimental Animal Center, Wuhan, China) were used in the study. The rats were randomly assigned to the sham-operated/no rTMS/no antagomir group (Sham, n = 15), the MCAO/no rTMS/no antagomir group (MCAO, n = 20), the MCAO/rTMS/no antagomir group (rTMS, n = 40), the MCAO/rTMS/antagomir group (Ant-25, n = 25) and the MCAO/rTMS/scrambled control group (Scr, n = 25). All groups underwent tMCAO surgery, with the exception of the Sham group. Moreover, apart from the MCAO group and the Sham group, the remaining groups received rTMS treatment. The experimental schedules are depicted in Figure 1.

### ICV Injection Protocol

Rats in the antagomir group and the scramble-miR group were subjected to the right-hemispheric intracerebroventricular (ICV) injection of antagomir (locked nucleic acid 3′ cholesterol-conjugated oligonucleotides, Ribobio Co, Guangzhou, China) or of scramble-miR (a non-targeting scrambled sequence as a negative control of antagomir, Ribobio Co, Guangzhou, China), respectively[16], [17]. The coordinates for the ICV injections (from Bregma) were as follows: AP = −0.8 mm, ML = −1.5 mm, and DV = −4.8 mm [18]. Ten microliters of antagomir-25 (Ant-25) or scramble-miR (Scr) was injected into the lateral ventricles just before tMCAO, with the various concentrations and effective miRNA depletions confirmed by the measurement of miR-106b∼25 levels via quantitative real-time PCR (qRT-PCR). Both Ant-25 and the corresponding Scr were dissolved in sterile saline solution and were continuously infused into the ipsilateral lateral ventricles conventionally.

### tMCAO Procedures

The rats were anesthetized with 10% chloral hydrate (400 mg/kg, i.p.). The right middle cerebral artery was occluded for 90 minutes (tMCAO) with subsequent reperfusion according to the method of Longa et al. [19]. Surgery was induced by the intraluminal suture occlusion method as previously described in detail [20]. Each rat was allowed to recover from the anesthesia and was returned to its cage with ad libitum access to food and water after the wound was sutured. During the surgical procedures, rectal temperature was maintained at 37 ± 0.5°C with a heat lamp. For the sham-operated group, only the external carotid artery was ligated.

### Neurobehavioral Evaluation

Evaluation in terms of neurological severity scores (NSSs) [21] was performed in all MCAO rats by the same person who was blind to the grouping of the rats. Assessment in terms of NSSs consists of tests of movement, sense of touch, reflexes and balance. The measures were rated on a 0-to-18 scale, with normal listed as 0 and the maximal deficit as 18. Only the rats (excluding those from the sham-operated group) scoring between 9 and 13 on the first postoperative day were included. Tests were performed during the light cycle and in the same sequence for all animals.

### rTMS Treatment

rTMS was delivered using a customized magnetic stimulator (YRD-CCI, Wuhan, China) and a round prototype coil (6 cm in diameter with 3.5 T peak magnetic welds) was applied during the treatment. Major features of rTMS coils are the depth of penetration and the focality of electric field, which are mutually exclusive [22]. To examine the effect of rTMS on the proliferation of adult neural stem cells (NSCs) in ipsilateral SVZ and allow the delivery of high-level energy to depth, we used the circular coil to conscious rats every 24 h for a 7-day period after tMCAO [23]. The circular coil, positioned perpendicular to the cortex approximately 0.5 cm to the right of the bregma, was initially moved craniocaudally (± 0.5 cm relative to the bregma) to optimize motor-evoked potential (MEP) responses in our experiment. The stimulation targets the primary motor cortex (M1) and could approximately reach a depth of 1 cm of the center regions under the coil. The rTMS treatment protocol consisted of stimulation for 3 s followed by rest for 50 s, which was repeated ten times (300 pulses per day) at the rate of 10 Hz. The stimulation intensity was set at 120% of the average resting motor threshold (RMT), namely 26% of the maximum output of the stimulator.

### Administration of Bromodeoxyuridine

Bromodeoxyuridine (BrdU) (50 mg/kg in saline, Sigma-Aldrich, USA) was intraperitoneally administered to the model group, the sham-operated group, the rTMS group, the antagomir group and the scramble-miR group three times for 12 h following the last rTMS treatment, and rats were sacrificed within 4 h after the last administration.

### Assessment of Experimental Focal Cerebral Ischemia of tMCAO Model Rats

Histological staining was performed 1 day after tMCAO, using 2, 3, 5-triphenyltetrazolium chloride (TTC, Sigma-Aldrich, USA) to define the overall degree of experimental focal cerebral ischemia due to the tMCAO of the model rats [24]. Five brains were selected randomly from the model group and were immediately frozen for 20 min, after which coronal sections were taken from the frontal pole. Afterwards, the sections were immersed in 1% TTC and then post-fixed with 4.0% paraformaldehyde. Finally, infarct areas in the sections emerged. The stained sections were then photographed and the infarct volumes were determined using ImageJ software.

### Tissue Preparation

To avoid potential impacts on the final results, we excluded rats that had exhibited abnormal behavioral effects during the treatment from the groups. All rats injected with BrdU were deeply anesthetized with 10% chloral hydrate (400 mg/kg, i.p.) and transcardially perfused with saline, followed by 4% paraformaldehyde in 0.1 M phosphate-buffered saline (PBS). The brains were removed and postfixed in the same fixative at 4°C overnight and were immersed consecutively in 20% and 30% sucrose at 4°C until they sank; then, 30-µm-thick consecutive coronal sections (0.3∼1.2 mm behind the bregma) were prepared. All other rats were immediately sacrificed and their ipsilateral tissues of cerebral infarction, including cortex and striatum [25] were dissected for qRT-PCR and western blotting.

### Immunofluorescence Staining and Image Processing

For immunofluorescence staining, sections were first rinsed in 0.1 M/L PBS (3×5 min). Afterwards they were incubated in blocking solution (10% normal donkey serum and 0.3% Triton-X100 in PBS) at room temperature (RT) for 2 h and 4°C for 24 h in mouse monoclonal Nestin antibody (1:100; BD Pharmingen, San Diego, CA, USA). The sections were then washed with PBS and incubated at RT while being protected from light for 3 h with donkey anti-mouse IgG (1:400; Life technologies, Jackson ImmunoRsearch, USA). For BrdU staining, DNA denaturing was required. After rinsing, the sections were immersed in 2N HCl at 37°C for 0.5 h. Then, sections were blocked in PBS containing 10% normal goat serum and 0.3% Triton-X100 at RT for 2 h. Primary rat monoclonal anti-BrdU (1:100; Abcam, UK), diluted in blocking buffer, was added to individual sections, which were incubated overnight at 4°C. The next day, the sections were again rehydrated with PBS and incubated in secondary antibody (goat anti-rat, 1:400; ZSGB-Bio, Beijing, China) at RT for 3 h. The stained slides were dehydrated, cover slipped with anti-quenching agent (p-phenylenediamine, PPD) and analyzed with a confocal laser scanning microscope (Olympus, Tokyo, Japan). Two regions in the ipsilateral SVZ were observed per rat with a 40× objective lens using microscopy, and the average values were recorded. A 488 nm diode laser and a 543 nm diode laser were used for the detection of BrdU^+^/Nestin^+^ positive cells. Z-stack images were captured with multiple images, each separated by a stepwise depth of 2 µm along the z-plane. The number of positive cells was counted in a blinded fashion from the digital images without image modification using an OLYMPUS FV10-ASW Viewer. Digital images to be represented were slightly modified to optimize image resolution, brightness and contrast using OLYMPUS FV10-ASW Viewer to best represent the immunofluorescence observed.

### Quantitative Real-Time PCR Analysis

MiRNA extraction from the ipsilateral cortical tissues was carried out using the Trizol reagent (Invitrogen, Carlsbad, CA, USA). For the quantification of miR-25, miR-93, and miR-106b, RNA was reverse transcribed using the TaqMan MicroRNA Reverse Transcription Kit and miRNA-specific stem-loop primers (Applied Biosystems, Foster City, CA). SYBR Green/Fluorescein qRT-PCR Master Mix [TaKaRa Biotechnology (Dalian), Dalian, China] was used to conduct qRT-PCR with a 7500 real-time PCR system (Applied Biosystems) as reported by others [26]. The relative expression of miRNA expression data was normalized to U6 RNA by2^−ΔΔCT^.

### Western Blotting Analysis

The ischemic ipsilateral SVZs of the rats were resected for western blotting. Protein was extracted through a serial procedure that involved the addition of protein extraction solution, homogenization by the vortexing of harvested tissues for 5 min, and harvesting of the supernatant after centrifugation for 10 min at 15,000 rpm at 4°C. Then proteins (50 µg-protein equivalents each) were electrophoresed on polyacrylamide gels, transferred to PVDF, and blocked with TBST containing 5% fat-free milk for 2 h. Primary antibodies diluted with TBST were poured onto the PVDF overnight at 4°C. Anti-rabbit p21 (1:500; Bioworld, Wuhan, China), anti-rabbit p57 antibody (1:700; Proteintech Group, Inc, Chicago, USA) and anti-goat PTEN (1:300; Santa Cruz, Inc, CA, USA) were used. After being washed five times with TBST for 5 min each time, secondary antibodies conjugated with HRP (1:40000; Bioworld, Wuhan, China) were added to PVDF for 2 h. Finally, the PVDF was washed again with TBST for 30 min, after which ECL (Thermo, USA) western blotting detection reagents were added to it. Images were taken with an X-ray film processor. Normalization was performed using mouse monoclonal GAPDH antibody (1:500; Santa Cruz, Inc, CA, USA). Quantitation of bands was undertaken using Gel-Pro Analyzer 4.0 software (Media Cybernetics, USA).

### Statistical Analysis

Data are presented as the mean±SEM and were analyzed using SPSS 17.0 (IBM Corporation, Somers, NY, USA). Statistical comparisons of results were performed by one-way ANOVA. The Bonferroni correction was taken to account for multiple testing. P<0.05 was regarded as statistically significant, and all numerical analyses were performed using the Graph Pad Prism program.

## Results

### Evaluation of the experimental focal cerebral ischemia of tMCAO model rats

**Figure 2:**
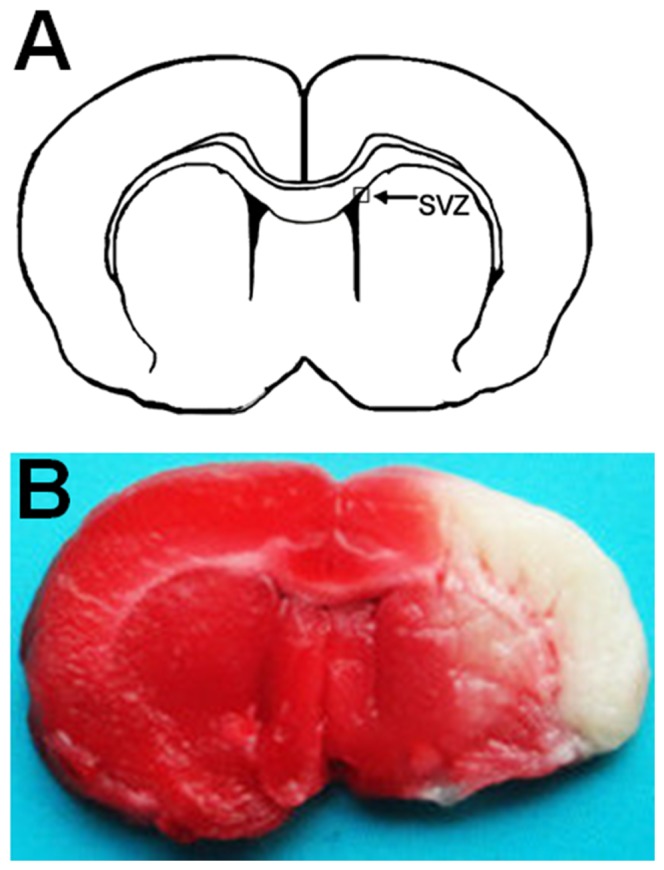
Infarct volume assessed by TTC staining 1 day after the tMCAO. (A) Position of SVZ in the coronal section of brain. Areas imaged for immunofluorescence studies are indicated by box. (B) Coronal brain section stained by TTC 1 day after tMCAO. The white areas without deep red-staining indicate ischemic areas. SVZ, subventricular zone.

The average size and location of the cerebral infarcts were critical for the evaluation of experimental focal cerebral ischemia. The total infarct volume after TTC-staining (177.63±22.54 mm^3^) mainly focused on the cerebral cortex and the striatum. In rats subjected to 90 min of tMCAO (n = 5), areas of infarction were clearly defined with TTC staining techniques (Figure 2).

### rTMS improved outcomes of neurological functions

**Figure 3:**
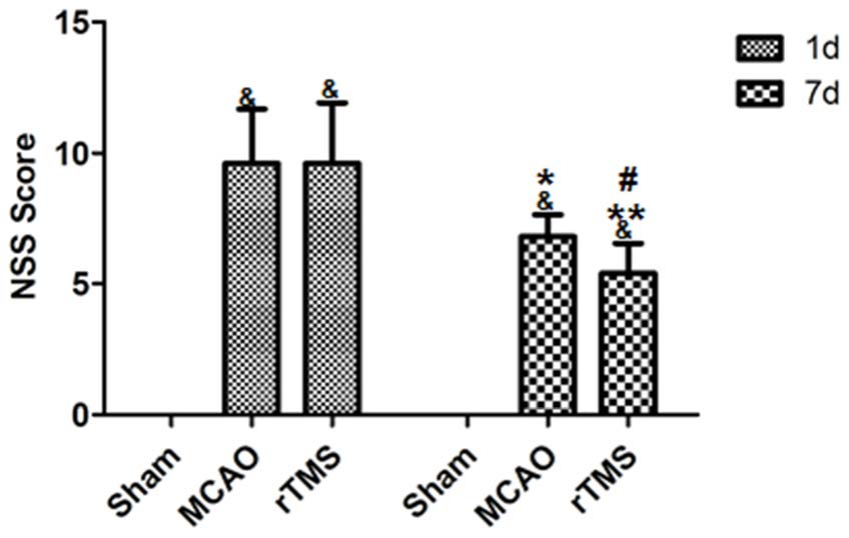
Neurobehavioral function was improved by rTMS after cerebral ischemia. NSSs were improved in MCAO rats treated with rTMS as compared with other groups. Data are presented as mean±SD. ^&^P<0.001 versus Sham group. ^*^P = 0.005 for MCAO group between day 7 and day 1. ^**^P<0.001 for rTMS group between day 7 and day 1. ^#^P = 0.019 between rTMS group and MCAO group 7 days after surgery.

To examine whether rTMS could improve the neurological function when subjected to tMCAO, we used the Neurological Severity Scores (NSSs) test. No differences in NSSs were observed among all groups before MCAO and animals subjected to MCAO showed severe behavior deficits 1 day after ischemia. Figure 3 shows that the score of sham-operated rats was 0 and the there was no significant difference in average scores among ischemic rats (p>0.05). There was a progressive improvement in NSSs in ischemic rats 7 days after surgery (P<0.001). However, rats in rTMS group had a significantly lower NSSs at the 7^th^ day after surgery, compared with the MCAO group (P = 0.019) (Fig. 3).

### rTMS promoted the proliferation of adult NSCs in the SVZ after focal cerebral ischemia

**Figure 4:**
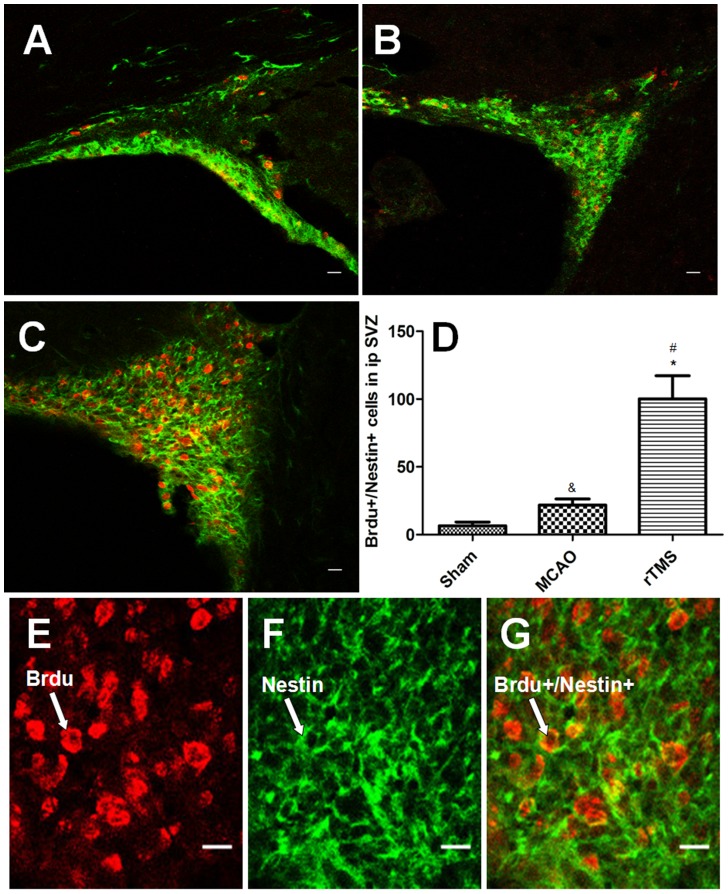
Confocal image of Brdu (red) and Nestin (green) co-immunofluorescence staining in the ipsilateral SVZ 7 days after surgery. Panels A–C show staining of Brdu^+^/Nestin^+^ positive cells in the ipsilateral SVZ from Sham (A), MCAO (B), and rTMS (C) groups (Bar = 20 µm). (D) Quantification analysis of the number of Brdu^+^/Nestin^+^ positive cells in the ipsilateral SVZ 7 days after surgery. Brdu positive cells were labeled red (E), Nestin positive cells were labeled green (F) and Brdu^+^/Nestin^+^ positive cells were double-labeled (G), Bar = 20 µm. ^#^ **P<**0.001 for rTMS group versus Sham group; ^*^ **P** = 0.001 for rTMS group versus MCAO group. ^&^ **P** = 0.044 for MCAO group versus Sham group. SVZ, subventricular zone.

Brdu, a thymidine analog that binds to DNA during S phase [27], is a specific maker for proliferation and Nestin is a specific marker for NSCs; therefore, immunofluorescence staining of Brdu and Nestin was employed to determine the proliferation of adult NSCs. By utilizing Brdu^+^/Nestin^+^ positive cells, adult NSC proliferation in the ipsilateral SVZ for the model group, the sham-operated group, and the rTMS group (n = 5 for each group) was examined to study the effect of rTMS in the present study. A significant increase in double positive cells was observed in the rTMS group and the model group relative to the sham-operated group. Moreover, Brdu^+/^Nestin^+^ cells in the rTMS group were 3.6 times more numerous than in the model group (Figure 4). These data indicated that focal cerebral ischemia did induce adult NSC proliferation in the SVZ and that the rTMS treatment facilitated such proliferation.

### rTMS altered the expression of miR-25 in the ischemic cortex

**Figure 5:**
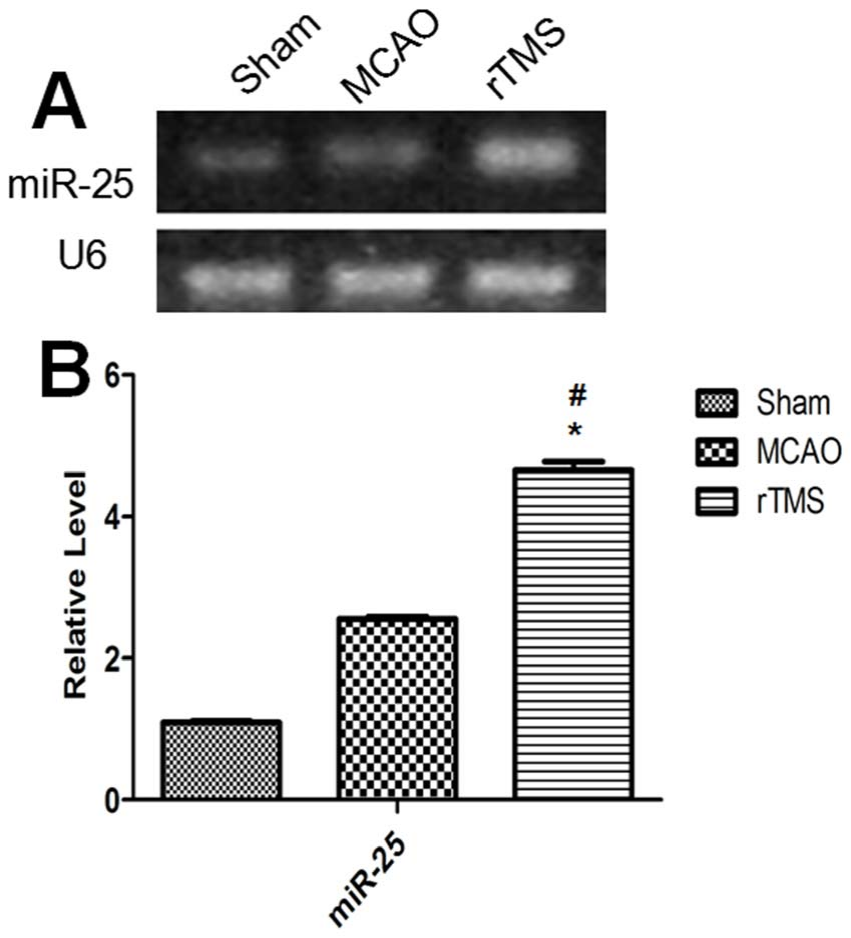
Expression changes of miR-25 in the ipsilateral cortex 7 days after surgery. (A) Electrophoresis of miR-25 and U6 on gel. (B) Relative expressions of miR-25 in different groups. ^#^ **P**<0.001 compared to the Sham group; ^*^ **P**<0.001 compared to the MCAO group.

To examine possible changes of miR-25 in response to the rTMS treatment after tMCAO, levels of its expression were determined by qRT-PCR in the ipsilateral cortex for the model group, the sham-operated group, and the rTMS group (n = 5 for each group). All data were derived by the calculations of 2^−ΔΔCT^ and were normalized to the expression of U6. The results revealed that (1) miR-25 levels in the model group were up-regulated 2.3-fold relative to the levels in the sham-operated group (2.55 versus 1.09, p<0.001), and (2) the miR-25 expression level in the rTMS group was also significantly up-regulated 4.3-fold relative to the sham-operated group (4.65 versus 1.09, p<0.001) and 1.8-fold relative to the model group (4.65 versus 2.55, p<0.001). These data suggested that tMCAO slightly induced the up-regulation of miR-25 and that 10 Hz rTMS prominently strengthened this effect (Figure 5).

### Effects of rTMS on miR-25 regulation of the expression of target genes p57 and PTEN

**Figure 6:**
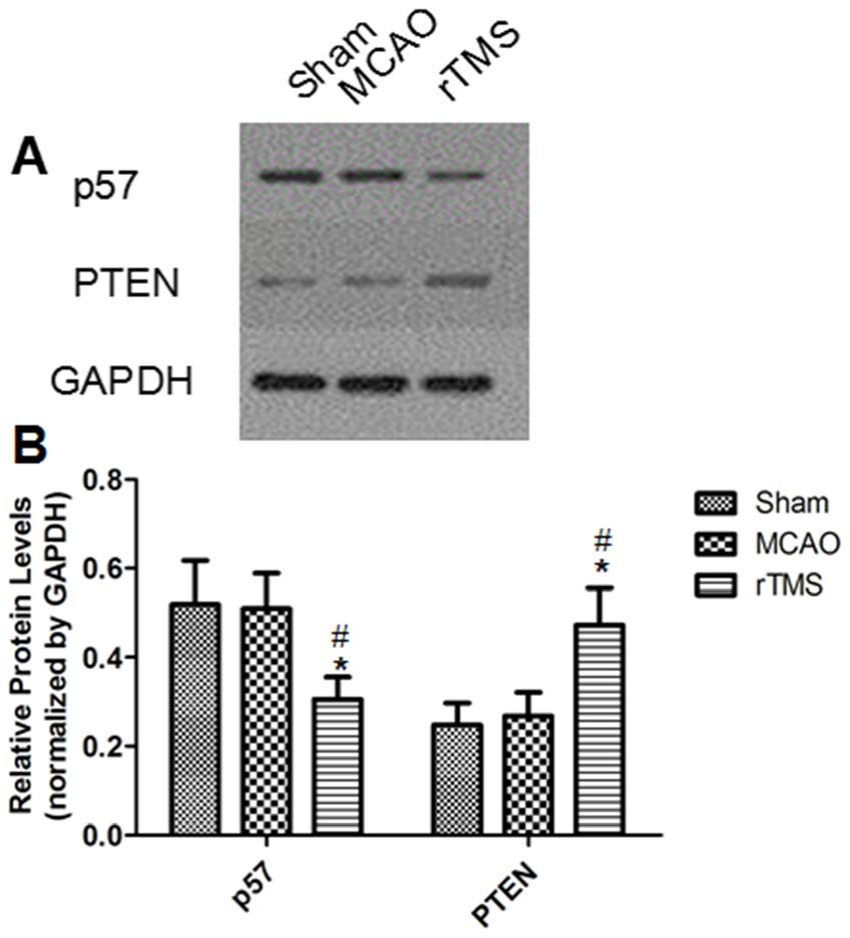
Expression changes of p57 and PTEN in the ipsilateral cortex 7 days after surgery. (A) Electrophoresis of p57, PTEN and GAPDH on gel. (B) The ratio of the target genes to GAPDH in different groups. ^#^ **P** = 0.005 for p57 compared to the Sham group; ^#^ **P** = 0.004 for PTEN compared to the Sham group; ^*^ **P** = 0.007 for PTEN compared to MCAO group.

The consequent changes of p57 and PTEN, which can generate adult NSC proliferation, were observed in the model group, the sham-operated group, and the rTMS group (n = 5 for each group) to further verify the effects of rTMS on the miR-25 cluster and to demonstrate a direct interaction between the target genes and the candidate miRNAs. The relative expression of p57 in the rTMS group appeared prominently suppressed compared to that in the other two groups (**p**<0.01). However, the relative quantity of PTEN expressed in the rTMS group was significant increased compared with other two groups (**p**<0.01), which implied that PTEN may play a complicated but not a dominant role in promoting adult NSC proliferation caused by rTMS. All relative expressions of those target genes between the sham-operated group and the model group failed to achieve statistically significant differences (**p**>0.5) (Figure 6).

### Depletion of miR-25 using antagomirs *in vivo*

To determine whether miR-25 plays a pivotal role in rTMS's promotion of adult NSC proliferation after focal cerebral ischemia, ICV injections of Ant-25 and Scr were used at different doses just prior to tMCAO, followed by 7 days of rTMS treatment. The rats were randomly assigned to the rTMS group (n = 15), the Ant-25 group (n = 15) and the Scr group (n = 15). Rats in each group were distributed equally according to doses of ICV. To verify their efficacies and specificities, the effects of Ant-25 and Scr on the levels of other members of the miR-106b∼25 cluster were also examined. In addition, the efficacy and specificity of the antagomir-induced silencing of miRNA in vivo were investigated by employing various doses.

**Figure 7:**
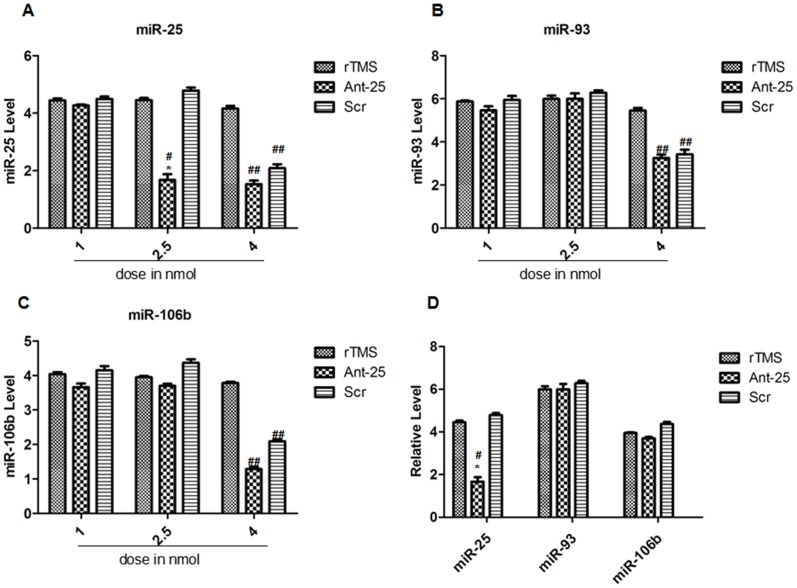
Effects of Ant-25 and Scr on miR-106b∼25 in the ipsilateral cortex 7 days after surgery. (**A**–**C**) **A**) miR-93, **B**) miR-106b and **C**) miR-25 levels in the ischemic cortex after ICV injection of 1 nmol, 2.5 nmol and 4 nmol of Ant-25 or Scr. (D) qRT-PCR measurement of the miR-106b∼25 cluster in the ischemic cortex after ICV injection of 2.5 nmol of Ant-25 or Scr. ^#^ **P**<0.001 for 2.5 nmol compared to the rTMS group; ^*^ **P**<0.001 for 2.5 nmol compared to the Scr group. ^##^P<0.001 for 4 nmol compared to the rTMS group.

The results showed that the levels of miR-25 were maintained at lower levels for 7 days after the injection of Ant-25, although miR-25 was not totally inhibited. A dose of 2.5 nmol Ant-25 significantly reduced miR-25, whereas 1 nmol had no effect (Figure 7A, D), and neither dose of Scr had an effect on miR-25 levels (Figure 7A, D). However, increasing the injection amount to 4 nmol appeared to produce off-target knockdown of miRNAs. Moreover, injection of 1 nmol of Ant-25 or Scr did not alter expression of miR-106b and miR-93 (Figure 7B, C), whereas at the higher 4.0 nmol dose, all levels of miR-106b∼25 were significantly reduced (Figure 7B, C).

### The injection of antagomir-25 up-regulated the expression of target gene p57

**Figure 8:**
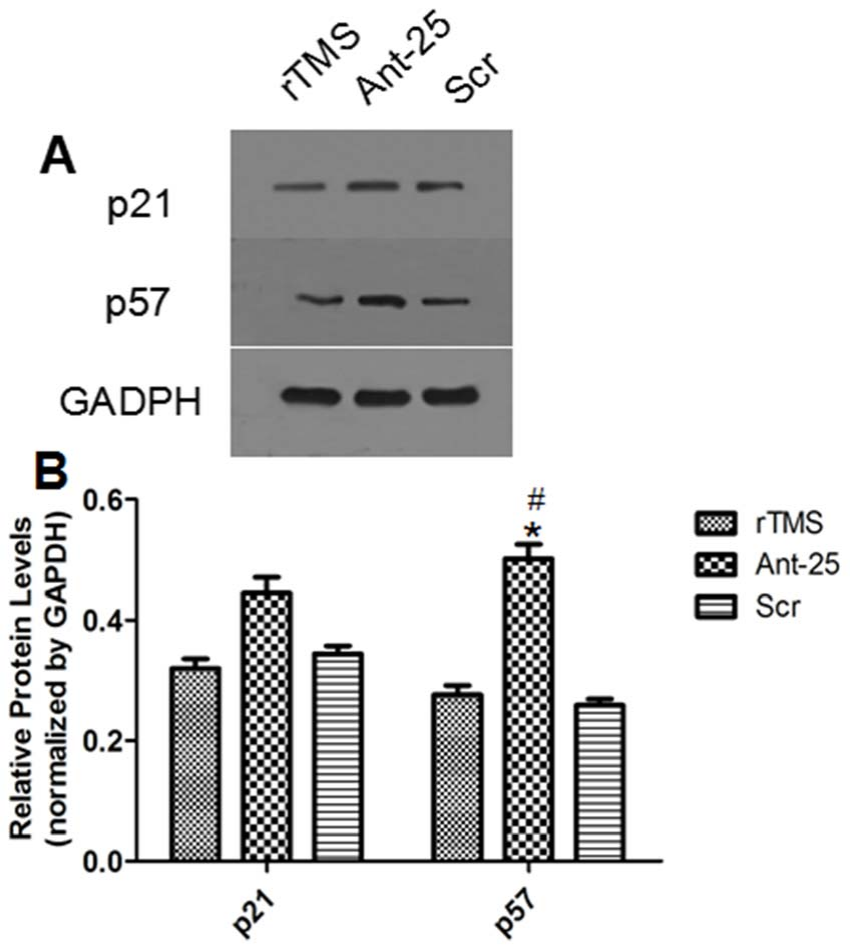
Effects of Ant-25 and Scr on p21 and p57 in the ischemic cortex 7 days after surgery. (A) Electrophoresis of p21, p57 and GAPDH on gel. (B) Relative levels of p21 and p57 protein from the different groups. ^#^ **P** = 0.018 compared to the rTMS group; ^*^ **P** = 0.012 compared to the Scr group.

The effect of ICV injection of Ant-25 treatment on the upregulation of miR-25 target gene p57 was demonstrated by Western blotting analysis. The rats were randomly assigned to the rTMS group (n = 5), the Ant-25 group (n = 5) and the Scr group (n = 5). After Ant-25 was introduced, the results revealed that the protein level of p57 was remarkably up-regulated in the ipsilateral cortex, confirming the direct suppression of p57 by miR-25. Moreover, the level of p21 was also examined to confirm specificity, and no difference was detected (Figure 8).

### Deletion of miR-25 blocked the proliferation of adult NSCs in the SVZ induced by rTMS

**Figure 9:**
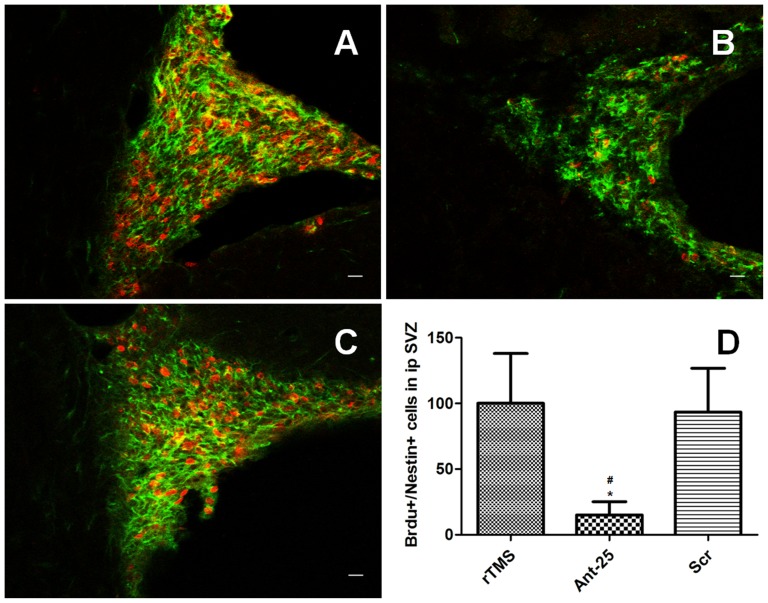
Antagomir-25 injection resulted in decreased proliferation of NSCs 7days after tMCAO. Panels A–C show staining of Brdu^+^(red)/Nestin^+^(green) positive cells in the ipsilateral SVZ from rTMS (A), antagomir-25/rTMS (B), and control antagomir/rTMS (C) groups(Bar = 20 µm). (D) Quantification analysis of the number of Brdu^+^/Nestin^+^ positive cells in the ipsilateral SVZ at 7 days after tMCAO. ^#^ **P** = 0.006 versus rTMS group; ^*^ **P** = 0.013 versus Scr group. SVZ, subventricular zone.

An evaluation was performed to determine whether Ant-25 would block the proliferation of adult NSCs in the SVZ after rTMS treatment. The rats were randomly assigned to the rTMS group (n = 5), the Ant-25 group (n = 5) and the Scr group (n = 5). As shown previously, rTMS-specific adult NSC proliferation in the SVZ after tMCAO was found, and no meaningful difference was observed between the rTMS group and the Scr group. However, the number of Brdu^+^/Nestin^+^ positive cells in the SVZ decreased due to the injection of Ant-25. Thus, our results clearly illustrated that the suppression of miR-25 blocked adult NSC proliferation after tMCAO (Figure 9).

## Discussion

rTMS, a novel neurological tool for its therapeutic benefits in recovery from stroke, is a promising approach for treating focal cerebral ischemia [28], [29]. One principal finding of our study was that 10 Hz rTMS could improve functional recovery and enhance neurogenesis in the adult SVZ after transient focal ischemia. MicroRNAs (miRNAs) are endogenous, short RNA sequences that have been proposed over the past decade to be involved in neurogenesis. In this paper, we reported that miR-25 is a crucial participant in promoting the effects of rTMS on adult NSC proliferation after focal cerebral ischemia in rats.

The standard treatment protocols of rTMS for stroke remain elusive. Preliminary studies have shown beneficial effects with different doses. Although the correlation between the total amount of stimuli and cortical excitability are not clear, generally, the amount of high-frequency stimulation is more than that of low frequency stimulation and the amount of supraliminal stimulation is higher than subthreshold stimulation when treatment is effective [30]. The rTMS applied with 10 Hz frequency at intensity lower than the motor threshold on the leisoned hemisphere improved the motor function after 160 applications of rTMS [31]. Besides, 10 Hz rTMS with 3000 pulses per day could also achieve improvement in motor performance in patients [32]. Moreover, when 25 Hz rTMS was delivered to cortical targets at 300 pulses and 100% motor threshold, therapeutic efficacy could persist [33]. In view of the efficacy and feasibility, we adopted a total stimulation amount of 300 pulses per day in the experiment.

There are two kinds of rTMS in terms of the frequency used: low frequency stimulation (∼1 Hz or <5 Hz) and high frequency stimulation (∼10 Hz or >10 Hz) [34]. Some investigators favor low-frequency rTMS over high-frequency one in that the low-frequency of rTMS is better tolerated and involves fewer risks. However, mounting evidence demonstrated that high-frequency rTMS, when applied to the affected hemisphere with stimulation parameters by strictly following the safety guidelines [35], was both safe and effective [36]. In clinical studies, the frequency used in high frequencies rTMS applied to ipsilateral cortex commonly ranged from 3 Hz to 10 Hz [37]. A number of studies applying 10 Hz rTMS to the leisoned hemisphere with intensity under the motor threshold reported substantially improved motor function [38], [39]. Our results were overall consistent with the findings of prior studies concerning the effect of high frequency rTMS on neurogenesis. Some researches reported that 15 Hz at an intensity of 100% RMT and 25 Hz at 70% RMT rTMS could increase hippocampal neurogenesis [5], [40]. Although much higher rTMS frequencies applied to ipsilateral motor area have been proved to be safe [41], [42], too high frequencies are also associated with greater risk for adverse events [43]. In the current study, we used 10 Hz frequency and few rats exhibited noticeable behavioural changes after rTMS, possibly due to low intensity used in our treatment. Besides, our result that NSC proliferation was increased in SVZ, confirmed the safety and efficacy of 10 Hz rTMS for the promotion of neurogenesis.

The proliferation of adult NSCs was enhanced in the ipsilateral SVZs of the tMCAO rats, as was evident from the results of the BrdU^+^/Nestin^+^ positive cells immunofluorescence staining. To our knowledge, this is the first report exploring the effect and mechanism of rTMS on adult NSC proliferation in a cerebral ischemic model from a miRNA-focused perspective. Our results are in line with previous studies indicating that focal cerebral ischemia led to marked increases in adult NSC proliferation [44], [45] and that rTMS treatment played a positive role in neurogenesis [46]. The experimental evidence of rTMS-induced adult NSC proliferation in the SVZ and other studied actions of rTMS (with regard to changes in neurotransmitter release, transsynaptic efficiency, signaling pathways and gene transcription as well as the secretion of neuroprotective molecules and neuronal viability) [47] underscore the potential offered by rTMS as a new strategy for neural regeneration after focal cerebral ischemia [48].

Despite the potential of rTMS, its mechanism of facilitating neurogenesis post-stroke is still controversial. rTMS could activate many regions of the cortex, but the most frequently examined cortical location is the motor cortex as its stimulation produces a well-quantified response, namely muscle contraction, which can be electromyographically recorded [49]. However, not only would the motor cortical area be activated, but also its connections, such as striatum, were activated [50]. Because one sidewall of SVZ is composed of striatum, samples were also from part of dissected SVZ tissue and upregulated miRNA could exert effects on SVZ. Here, we observed that the expression level of miR-25 in the ipsilateral cortex was significantly higher after 10 Hz rTMS than for the model group without rTMS. Meanwhile, the quantity of target gene p57 was distinctly decreased in a corresponding manner. Additionally, adult NSC proliferation has been suppressed in ischemic SVZs after 10 Hz rTMS treatment due to miR-25 knockdown accompanied by elevated expression of p57. Brett J O et al. carried out an elaborate study of the role of miR-106b∼25 in adult NSCs and noted that miR-25 knockdown decreases NSC proliferation and that miR-25 overexpression increases adult NSC proliferation [9]. Hence, based on our results and theoretical support, we can assert that 10 Hz rTMS appears to facilitate the proliferation of adult NSCs after cerebral ischemia by promoting the expression of miR-25.

As is well known, factors regulate the functions of adult NSCs largely by directing changes in gene expression, and miRNAs represent an additional layer of gene expression control for regulating adult NSCs [51], [52]. As previously mentioned, p57, co-operatively suppressed by miR-25, is a Cdk inhibitor (CKI) and mediates the transitions between cell-cycle phases by binding to Cdks. The Cip/Kip family of proteins blocks the progression through all stages of G1/S, thereby functioning as a ‘brake on the cell cycle’. Combined with the other results, we provide novel evidence that miR-25-dependent regulation of p57 influences the proliferation of adult NSCs induced by rTMS after focal cerebral ischemia. Taken together, the activation of miR-25 could promote a proliferation state of adult NSCs through p57, and these findings lead us to conclude that rTMS mainly induces the activation of the miR-25/p57 pathway, thereby promoting adult NSC proliferation after focal cerebral ischemia.

In addition, the current study also reveals an interesting effect of different doses of antagomir-25 and its scrambled antagomir on the miRNA levels of miR-25 and the unrelated miR-93 and miR-106b. Our data revealed that depletion of miR-25 had some relevance to ICV injection concentrations of Ant-25 and that excessive or inadequate doses of antagomirs had differential impacts on miRNA expression. Specifically, dosing the injected amount at 4 nmol appeared to produce off-target knockdown of the miRNAs, whereas 1 nmol seemingly had no effect. Similarly, previous reports on other tissues have also observed this phenomenon [53], [54]. Overall, our results provide further evidences verifying the efficacy and specificity of the antagomir-induced silencing of specific miRNAs in vivo.

Although the miR-25/p57 pathway was demonstrated to play a role in the therapeutic mechanisms of rTMS for focal cerebral ischemia in the present study, there may even be crosstalk between the different pathways that are focused on miR-25 or targeted by the other two members of the miR-106b∼25 cluster. For example, the expression of p57 is also regulated by the TGFβ pathway, primarily by downregulating the activity of the Cyclin-CDK complex in the G1 stage [55]. Thus, it is possible that miR-25 regulates adult NSCs by coordinately modulating networks, and further study is required to explore the full set of regulatory mechanisms of rTMS for cerebral ischemia.

## Conclusions

In summary, the present study demonstrated that 10 Hz rTMS can promote adult NSC proliferation in the SVZ after focal cerebral ischemia. The protective effect of rTMS was associated with the miR-25/p57-signaling pathway. These results indicate that rTMS could be a promising candidate for complementary therapy that facilitates self-repairing capabilities after focal cerebral ischemia.
